# Incidence and Seroprevalence of Avian Influenza in a Cohort of Backyard Poultry Growers, Egypt, August 2015–March 2019

**DOI:** 10.3201/eid2609.200266

**Published:** 2020-09

**Authors:** Mokhtar R. Gomaa, Amira S. El Rifay, Dina Abu Zeid, Mona A. Elabd, Eman Elabd, Ahmed Kandeil, Noura M. Abo Shama, Mina N. Kamel, Mohamed A. Marouf, Ahmed Barakat, Samir Refaey, Amal Naguib, Pamela P. McKenzie, Richard J. Webby, Mohamed A. Ali, Ghazi Kayali

**Affiliations:** National Research Centre, Giza, Egypt (M.R. Gomaa, A.S. El Rifay, D. Abu Zeid, M.A. Elabd, E. Elabd, A. Kandeil, N.M. Abo Shama, M.N. Kamel, M.A. Marouf, M.A. Ali);; Ain Shams University, Cairo, Egypt (A. Barakat);; Ministry of Health and Population, Cairo (S. Refaey, A. Naguib);; St. Jude Children’s Research Hospital, Memphis, Tennessee, USA (P.P. McKenzie, R.J. Webby);; Human Link, Hazmieh, Lebanon (G. Kayali);; University of Texas Health Sciences Center at Houston, Houston, Texas, USA (G. Kayali)

**Keywords:** avian influenza, Egypt, epidemiology, cohort, incidence, seroprevalence, viruses, respiratory diseases, vaccine-preventable diseases, zoonoses, poultry

## Abstract

Currently enzootic avian influenza H5N1, H9N2, and H5N8 viruses were introduced into poultry in Egypt in 2006, 2011, and 2016, respectively. Infections with H5N1 and H9N2 were reported among poultry-exposed humans. We followed 2,402 persons from households raising backyard poultry from 5 villages in Egypt during August 2015–March 2019. We collected demographic, exposure, and health condition data and annual serum samples from each participant and obtained swab samples from participants reporting influenza-like illness symptoms. We performed serologic and molecular analyses and detected 4 cases of infection with H5N1 and 3 cases with H9N2. We detected very low seroprevalence of H5N1 antibodies and no H5N8 antibodies among the cohort; up to 11% had H9 antibodies. None of the exposure, health status, or demographic variables were related to being seropositive. Our findings indicate that avian influenza remains a public health risk in Eqypt, but infections may go undetected because of their mild or asymptomatic nature.

For more than a decade, Egypt had endemic avian influenza viruses (AIVs) that infected humans and caused substantial economic losses in the poultry industry (*1*). Co-circulation of highly pathogenic avian influenza (HPAI) H5N1 and H5N8 viruses and low pathogenic avian influenza (LPAI) H9N2 viruses among poultry was observed (*2*). The widespread circulation of various AIV subtypes in domestic poultry resulted in evolutionary changes that affected several virus characteristics (*3*–*6*).

According to the World Health Organization, the number of confirmed human H5N1 cases in Egypt is 359, of which 120 were fatal (*7*). Those cases were detected by public health surveillance when the patients were admitted to hospitals with influenza-like illness (ILI) and had a history of poultry contact (*8*). However, this number is likely an underestimate because many patients might have mild symptoms or be asymptomatic and are less likely to seek medical care and therefore would not be tested or reported. Serologic testing might be a better tool in understanding the actual prevalence of disease because it can identify patients with mild or asymptomatic infections (*9*). A 3-year seroprevalence study showed that ≈2% of persons in Egypt exposed to poultry had been infected with H5N1 (*10*). This study demonstrated that the number of cases is underreported and that the case-fatality rate is consequently overestimated. Another study conducted in Beheira Governorate, located between the cities of Alexandria and Cairo and through which substantial transport between those 2 urban centers occurs, showed that the seroprevalence rate for H5 antibodies was 4% in poultry workers (*11*,*12*).

H9N2 viruses circulating in poultry in Egypt have human-like rather than avian-like receptor specificity (*6*). The clinical symptoms of H9N2 infection in humans are always mild, which complicates detection of human cases through hospital-based surveillance systems (*13*). Four laboratory-confirmed human cases of H9N2 infection were reported to the World Health Organization from Egypt during March 2015–April 2016. Seroprevalence of H9N2 antibodies in persons in Egypt exposed to poultry were 5.6% and 7.5% during the first 2 years of introduction of H9N2 in Egypt (*10*).

Since 2017, several H5N8 outbreaks of clade 2.3.4.4 (group B) have been detected in domestic poultry in several governorates in Egypt (*2*,*14*). No human cases of H5N8 were reported in Egypt or elsewhere.

Serologic studies and hospital-based surveillance do not provide accurate estimates of incidence of avian influenza infections in exposed humans. Hence, we designed a household prospective cohort study to examine the incidence, human-to-human transmission, and prognosis of AIV infections among poultry-exposed growers in Egypt.

## Methods

### Study Design

Details of the study design and protocol were previously published (*15*). In brief, households raising backyard poultry were selected from 5 villages in 4 Nile Delta governorates (Sharkiya, Gharbiya, Kafr El Sheikh, and Qalyubiya) and Fayyoum Governorate starting in August 2015. Baseline enrollment was completed in March 2017. Follow-up period 1 occurred April 2017–March 2018, and follow-up period 2 occurred April 2018–March 2019. All persons within the household who were >2 years of age were invited to participate. Household data pertaining to raising poultry was collected. Individual demographic, poultry exposure, and health condition data were collected. At the baseline and follow-up period 1, a serum sample was collected from each participant.

As of April 2017, study staff were visiting enrolled households on a weekly basis to check whether any study participant was reporting ILI symptoms. ILI was defined as having fever of >38°C as well as cough, sore throat, or both. The frequency of household visits was increased to twice per week with the start of the influenza season, which typically occurs October–March in Egypt. When a study participant was verified to have ILI symptoms, a serum sample was collected, and nasal and an oropharyngeal swab specimens were obtained and tested by reverse transcription PCR (RT-PCR) for influenza A infection. If any of the samples tested positive for influenza A, the person was considered an index case-patient, and the study team obtained nasal and oropharyngeal swab specimens from the participant on days 3, 6, 9, and 14 after the first swab day and a serum sample on day 14. Furthermore, all previously enrolled participants residing with the index case-patient were swabbed according to the same sampling schedule.

### Laboratory Methods

Reverse-genetics avian influenza viruses (rg A/chicken/Egypt/D10552B/2015 [clade 2.2.1.2 H5N1]) and (rgA/green-winged teal/Egypt/871/2016 [clade 2.3.4.4 H5N8] and A/chicken/Egypt/D10802C/2015 [G1-like H9N2]) were cultivated in 10 day-old, specific pathogen–free, embryonated chicken eggs and incubated for 48 h at 37°C, then chilled at 4°C for 4 h. The allantoic fluid was harvested, clarified, tested for the hemagglutinin (HA) gene and 50% tissue culture infective dose titer, and then frozen at −80°C until use.

Collected blood samples were kept on ice until they reached the laboratory on the same day. Serum was separated by centrifugation at 1,000 × *g*, aliquoted, and frozen at −20°C until use. Virus microneutralization assay was performed to test all collected serum samples for antibodies against the 3 AIVs (*16*). Chicken hyperimmune serum samples previously produced individually against the 3 viruses were used as positive controls and included in each assay. Neutralization capacity for each enrolled participant’s serum sample of infected MDCK cells was tested for hemagglutination activity of viruses by using 0.5% chicken red blood cells (RBCs) in an HA gene assay. The absence of hemagglutination was considered a positive test result for antibodies to the virus. Virus microneutralization assay positivity was considered at an endpoint titer of >1:80. Seasonal influenza A/Brisbane/10/07(H3N2) and pandemic A/California/04/09(H1N1) viruses were used to determine seroprevalence antibodies against both viruses by a hemagglutination inhibition (HI) assay, using 0.5% turkey RBCs (*17*). 

### Molecular Detection

Nasal and oropharyngeal swabs were subjected to viral RNA extraction by using the QIAamp Viral RNA Mini Kit (QIAGEN, https://www.qiagen.com), followed by detection of influenza A infection by M gene RT-PCR (*17*). Influenza A–positive samples were subtyped with RT-PCR using subtype-specific primers for HA gene subtyping (*18*). As soon as a participant had a positive M gene RT-PCR result, they were informed and advised to seek medical care. Amplicons of the appropriate sizes of positive HA gene subtypes were subsequently gel-purified by using the QIAGEN Gel Extraction Kit and then delivered for sequencing at a sequencing facility in South Korea (Macrogen, https://www.macrogen.com). Sequences were assembled by using SeqMan Lasergene 7 (DNASTAR, https://www.dnastar.com). Sequence alignments were performed by using BioEdit version 7.0 (https://www.biodeit.software.informer.com). The phylogenetic tree was constructed by using MEGA version 7 (https://www.megasoftware.net) by applying the neighbor‐joining method with the Kimura 2‐parameter substitution model and 1,000 bootstrap replicates.

### Statistical Analyses

SPSS Stastistics 23 (IBM, https://www.ibm.com) was used for statistical analyses. Statistical differences between proportions were tested by using the Pearson’s χ^2^ test or Fisher exact test. The Student *t*-test was used to compare continuous variables. A p value <0.05 was considered statistically significant.

### Ethical Considerations

This study was approved by the institutional review boards of Human Link and St. Jude Children’s Research Hospital and by the Ethics Committee of the National Research Centre. Written informed consent was obtained from adults >18 years of age. Informed assent and parental approval were obtained for participants 14–17 years of age. Oral assent and parental approval were obtained for participants 7–13 years of age. Parental approval was obtained for participants 2–7 years of age.

## Results

A total of 2,402 participants were enrolled from 390 households in the 5 study sites (Appendix Table). The median number of participants per household was 5 (range 1–20). Various domestic mammals and poultry were raised by the households, most frequently chickens and ducks. Approximately one third of the households raised pigeons and geese, whereas ≈12% raised turkeys (Table 1). Table 2 shows the distribution of poultry-raising practices followed by participants. Approximately 17% of households reported raising poultry within the household, whereas ≈53% kept the poultry on the roof and ≈30% in a barn. Dead poultry was disposed of by burying, discarding in a closed trash bag, or burning by 44% of the respondents, whereas 56% either dumped the carcasses in the open trash or in small water canals. Approximately 38% said that they would consult a veterinarian when they noticed ill poultry, and 14% said that they would isolate the ill birds from the rest of the flock. Approximately 33% of respondents said they would get rid of the sick bird while it was alive, whereas ≈12% said they would slaughter and eat it. One third of the respondents reported using avian influenza vaccines for their poultry, but only 1% of those were able to determine that the vaccine was used against H5N1 influenza virus, whereas the rest did not know what the vaccine was used against. Vaccination was mostly performed by a veterinarian (77%) or by a hired aid or a family member.

**Table 1:** Animals raised by enrolled households in a study of avian influenza among backyard poultry growers, Egypt, August 2015–March 2019

Animal	% Households	Median no. animals (range)
Chickens	91.3	15 (1–100)
Ducks	83.6	10 (1–700)
Pigeons	34.4	10 (1–100)
Geese	27	4 (1–20)
Donkeys	25.9	1 (1–3)
Buffaloes	25.6	2 (1–7)
Cows	24.6	1 (1–7)
Sheep	15.3	1 (1–30)
Goats	12.1	2 (1–30)
Turkeys	11.7	14 (1–15)
Dogs	8.2	2 (1–4)
Rabbits	7.8	5 (1–70)
Horses	5.8	1 (1–10)
Cats	2.5	7 (1–10)
Camels	0.01	1 (1–2)

**Table 2:** Poultry-raising practices reported by participants in a study of avian influenza among backyard poultry growers, Egypt, August 2015–March 2019

Characteristic	No. (%)
Where do you keep the poultry?	
On the roof	1,267 (52.7)
In a barn	716 (29.8)
Inside the house	419 (17.4)
What do you do with poultry carcasses?	
Bury	255 (10.6)
Place in a closed bag and throw in the trash	728 (30.3)
Burn	83 (3.5)
Throw in the trash without a bag	652 (27.1)
Throw in a water stream without a bag	684 (28.5)
What do you do if you suspect sick poultry?	
Nothing	81 (3.4)
Set loose away from the house	791 (32.9)
Seek veterinary advice	909 (37.8)
Quarantine away from the rest of the flock	332 (13.8)
Slaughter and consume the meat	289 (12.0)
Do you vaccinate poultry against avian influenza?	
Yes	902 (37.6)
No	1,500 (62.4)
Who administers the vaccine?	
Family member	155 (17.1)
Veterinarian	698 (77.4)
Worker	49 (5.4)

Demographic and health data of the participants are summarized in Table 3. Respondents’ age ranged from 2 to 102 years. The mean age was 25 years, and the median age was 21 years. The distribution of enrolled participants by sex, age (adults versus children), and marital status (single versus other) was almost the same. Most participants were uneducated or received only a primary education (68%), and 12% had a job as a skilled laborer or professional. Approximately 10% had chronic health problems, and ≈2% had long-term respiratory problems. Less than 10% reported currently using tobacco. Only 4 participants reported ever receiving the influenza vaccine.

**Table 3:** Demographic and health data of participants in a study of avian influenza among backyard poultry growers, Egypt, August 2015–March 2019

Characteristic	No. (%)*
Age group, y	
2–6	381 (15.9)
7–14	552 (23.0)
15–17	142 (5.9)
>18	1,322 (55.2)
Sex	
F	1,317 (54.9)
M	1,080 (45.1)
Educational level	
Not educated	822 (34.3)
Elementary	800 (33.4)
Intermediate	448 (18.7)
Vocational	35 (1.5)
Secondary	95 (4.0)
College	116 (4.8)
Graduate degree	78 (3.3)
Marital status	
Divorced	8 (0.3)
Married	1,048 (43.7)
Single, never married	1,232 (51.4)
Widowed	109 (4.5)
Occupation	
Toddler	339 (14.2)
Student	783 (32.8)
Housewife	698 (29.2)
Unskilled labor or unemployed	282 (11.8)
Skilled labor or professional	287 (12.0)
Chronic disease	
Yes	250 (10.4)
No	2,147 (89.6)
Long-term breathing problems	
Yes	45 (1.9)
No	2,340 (98.1)
Current tobacco user	
Yes	203 (8.6)
No	2,164 (90.3)
Ever received the influenza vaccine	
Yes	4 (0.2)
No	2,377 (99.8)

*Totals do not add up to 2,402 for all characteristics because of missing data.

The median number of days per week that the participants had direct contact with poultry was 6 days. The participants spent a median of 10 minutes per day in direct contact each time they came in direct exposure with the poultry. Table 4 summarizes the poultry exposure practices of the participants. More than 70% reported cleaning poultry cages or feeding poultry, whereas 30% reported not having any direct poultry exposure. Among those who had direct poultry exposure (cleaning or feeding), only 10% reported using a dedicated garment. Approximately one third of the participants reported slaughtering poultry. Slaughtered poultry were mostly kept in a dedicated barrel to bleed (80%), but 20% of respondents left that to occur in the open. Most of the respondents cleaned the used utensils after slaughtering, mostly by using soap and water. Slaughter waste was disposed in closed bags and thrown into the trash (46% of respondents), thrown in open trash (25%), or dumped into small canals (29%).

**Table 4:** Poultry exposure data of participants in a study of avian influenza among backyard poultry growers, Egypt, August 2015–March 2019

Characteristic	No. (%)*
What is the type of exposure you have with poultry?	
Cleaning area where poultry is kept	671 (28.0)
Feeding poultry	366 (15.3)
Only walk through area where poultry is kept	300 (12.5)
Play in area where poultry is kept	347 (14.5)
Do you usually slaughter poultry?	
Yes	694 (31.3)
No	1,526 (68.7)
Use of personal protective equipment while slaughtering poultry	
Yes (apron, boots, dedicated garment, face mask, gloves)	75 (10.8)
No	619 (89.2)
Where is slaughtered poultry kept to bleed?	
In a dedicated barrel	553 (79.6)
In a sink	45 (6.5)
On the floor inside the house	49 (7.1)
On the floor outside the house	47 (6.8)
Are tools cleaned after slaughtering?	
Yes	684 (98.6)
No	10 (1.4)
How are tools cleaned?	
Water only	103 (14.8)
Soap and water	539 (77.7)
Disinfectant	52 (7.5)
Use of personal protective equipment while defeathering poultry	
Yes (apron, boots, dedicated garment, face mask, gloves)	54 (7.8)
No	640 (92.2)
Method of disposing slaughtering waste	
Place in a closed bag and throw in the trash	299 (45.7)
Feed to other animals	1 (0.2)
Throw in the trash without a bag	163 (24.9)
Throw in a water stream without a bag	191 (29.2)

*Totals do not add up to 2,402 for all characteristics because of missing data or structure of the question.

Serologic findings at baseline and follow-up period 1 (2017–2018) are shown in Table 5. Serum samples were successfully collected from 2,397 persons at baseline and from 2,051 at follow-up period 1. Seroprevalence of H1N1 antibodies was ≈30% and of H3N2 antibodies was ≈50%. At baseline, 9 (0.4%) participants had H5N1 antibodies. Four participants had a titer of 1:80, and 5 had a titer of 1:160. Of the 9 participants, 7 came from 2 adjacent homes. At follow-up period 1, only 4 (0.2%) participants were seropositive for H5N1 antibodies, 3 with 1:80 titers and 1 with a 1:160 titer. None of the participants who were seropositive at baseline remained seropositive at follow-up period 1. At baseline, 266 (11%) participants had H9N2 antibodies. Of these, the 227 had 1:80 titers, 37 had 1:160 titers, and 2 had 1:320 titers. Household clusters of seropositive persons were observed for 223 participants, with cluster sizes ranging from 2 to 10 participants. At follow-up period 1, only 3 (0.1%) participants had H9N2 antibodies. All participants had a 1:80 titer and were from the same household, and only 1 of these participants was positive at baseline with the same titer. No participants had H5N8 antibodies at baseline or follow-up period 1.

**Table 5:** Seroprevalence of antibodies against influenza A virus subtypes H5N1, H5N8, H9N2, H1N1, and H3N2 in participants in a study of avian influenza among backyard poultry growers, Egypt, August 2015–March 2019

Period and influenza virus subtype	No. (%)
Baseline period, August 2015–March 2017	
H5N1 positive	9 (0.4)
H5N1 negative	2,388 (99.6)
H5N8 positive	0
H5N8 negative	2,397 (100.0)
H9N2 positive	266 (11.1)
H9N2 negative	2,131 (88.9)
H1N1 positive	656 (29.5)
H1N1 negative	1,569 (70.5)
H3N2 positive	1,115 (49.3)
H3N2 negative	1,148 (50.7)
Follow-up period 1, April 2017–March 2018	
H5N1 positive	4 (0.2)
H5N1 negative	2,046 (99.8)
H5N8 positive	0
H5N8 negative	2,046 (100.0)
H9N2 positive	3 (0.1)
H9N2 negative	2,043 (99.9)
H1N1 positive	612 (29.8)
H1N1 negative	1,439 (70.2)
H3N2 positive	1,034 (50.5)
H3N2 negative	1,015 (49.5)

*Totals do not add up to 2,402 at baseline period or 2,189 at follow-up period 1 because of missed serum sample collection or insufficient sample volume.

During follow-up period 1, a total of 400 participants (16.7% of the cohort) were confirmed to have ILI symptoms. Of these, 113 were positive for influenza A by RT-PCR (28% of those with ILI and 4.7% of cohort overall). Four case-patients were subtyped as infected with H5N1 virus by RT-PCR and confirmed by sequencing. The incidence of H5N1 infection in this cohort of 2,402 persons was 17 cases/10,000 exposed persons. Phylogenetic analysis revealed that the viruses causing the infection were of clade 2.2.1.2, which are unique to and endemic in Egypt.

The first case was in a 5-year-old boy with exposure to poultry at the household and at a live bird market. Only the swabs obtained on day 1 of the illness were positive for H5N1. The serum sample titer obtained on day 1 was <1:10, whereas a titer of 1:40 was detected on day 14. The boy’s symptoms included fever, cough, sore throat, myalgia, malaise, headache, runny nose, and diarrhea. Fever persisted for 4 days; cough and sore throat continued to occur throughout the 14-day follow-up period. No household contacts showed symptoms, virus shedding, or seroconversion.

Case 2 was in an 11-year-old girl with direct contact with chickens and ducks. Only the swabs obtained on day 1 of the illness were positive for H5N1. The girl’s serum sample titers were <1:10. Symptoms included fever, cough, sore throat, malaise, headache, and runny nose. Fever and sore throat persisted for 2 days; all other symptoms cleared by the fourth day. No household contacts showed symptoms, virus shedding, or seroconversion.

Case 3 was in a 5-year-old boy with direct contact with chickens and ducks. Swabs continued to be positive up to day 9 of sampling. The boy’s serum sample titers were <1:10. Symptoms included fever, cough, sore throat, malaise, headache, diarrhea, myalgia, and runny nose. Fever and cough persisted until day 9; all other symptoms were clear by the fourth day. No household contacts showed symptoms, virus shedding, or seroconversion.

Case 4 was in a 27-year-old woman who had direct contact with chickens and ducks. Swabs continued to be positive up to day 9 of sampling. The woman’s serum sample titers were <1:10. Symptoms included fever, cough, sore throat, malaise, headache, myalgia, and runny nose. Sore throat and cough persisted until day 12; all other symptoms were clear by the fourth day. No household contacts showed symptoms, virus shedding, or seroconversion.

During follow-up period 2, a total of 2,189 participants remained in the study, compared with 2,402 who participated in follow-up period 1. Of these, 740 (33.8%) participants were confirmed to have ILI symptoms, of whom 158 were positive for influenza A by RT-PCR (21% of those with ILI and 7.2% of the cohort overall). Four case-patients were infected with H9N2 and 1 with H5N1 virus according to RT-PCR results, which were confirmed by sequencing. The incidence of H9N2 infection was 18 cases/10,000 exposed persons. Incidence of H5N1 was 5 cases/10,000 exposed persons. Phylogenetic analysis revealed that the H9N2 viruses causing the infection were G1-like viruses (Appendix Figure 1), similar to viruses circulating in poultry in Egypt, whereas the H5N1 viruses were of clade 2.2.1.2 (Appendix Figure 2).

The H5N1 case was in a 10-year-old girl with direct contact with chickens and ducks. Swabs were positive on day 1 only. The girl’s serum sample titers were <1:10. Symptoms included fever, cough, sore throat, and myalgia. Sore throat and cough persisted until day 12; all other symptoms were clear by the fourth day. Two household contacts showed symptoms but were negative for H5, virus shedding, or seroconversion.

The first H9N2 case was in a 64-year-old woman with direct contact with chickens and ducks. Swabs were positive up to day 5 of sampling. The woman’s serum sample titers were <1:10. Symptoms included fever, cough, sore throat, diarrhea, runny nose, and myalgia, all of which persisted up to 8 days. No household contacts showed symptoms, virus shedding, or seroconversion.

The second H9N2 case was in an 8-year-old girl with direct contact with chickens and ducks. Swabs were positive up to day 5 of sampling. The girl’s serum sample titers were <1:10. Symptoms included fever, cough, sore throat, runny nose, and myalgia, all of which persisted up to 5 days except for coughing, which continued for 3 more days. No household contacts showed symptoms, virus shedding, or seroconversion.

The third H9N2 case was in a 15-year-old boy with direct contact with chickens and ducks. Swabs were positive up to day 5 of sampling. The boy’s serum sample titers were <1:10. Symptoms included fever, cough, sore throat, runny nose, and myalgia, all of which persisted up to 10 days. No household contacts showed symptoms, virus shedding, or seroconversion.

The fourth H9N2 case was in a 13-year-old girl. This case was detected 3 days after follow-up of another H3N2-positive case-patient in her household. Of the 5 members in her household, 4 had low-grade fever. H9N2 was detected in nasal and oral swab specimens from the case-patient, and the remaining household contacts were either negative or had H3N2 infection. The case-patient had low-grade fever, sore throat, runny nose, malaise, and breathing difficulty up to 6 days after detection.

## Discussion

We conducted a large prospective household cohort study of AIV infections among persons exposed to backyard poultry in Egypt. Our study design solved several problems that were noted by other similar studies (*19*,*20*). The larger sample size provided enough statistical power to detect the rare event of detecting active infection with AIVs. Following the households closely enabled us to detect those cases and document case-patients’ shedding, symptoms, and seroconversion. This approach also enabled us to verify whether human-to-human transmission was occurring.

Our epidemiologic findings confirm that backyard poultry raising practices have low to no biosecurity measures; these practices included keeping poultry within the household, disposing dead poultry in the open trash or water streams, and letting ill poultry loose. Growers did not frequently use personal protective equipment while exposed to poultry. Few of them reported using dedicated garments while tending to poultry, even though this measure was a main recommendation of previous educational campaigns. Similarly, only 10% of participants who reported slaughtering poultry (the riskiest behavior because of the aerosols generated [*21*]) used any personal protective equipment. Reviewing, revising, and updating health education and awareness campaigns based on scientific evidence might assist in decreasing the incidence of infection with AIVs in Egypt (*21*).

Very low seroprevalence of H5N1 antibodies was detected, whereas up to 11% of the participants had H9 antibodies, possibly because during the study period H5N1 infection was rare in poultry but H9N2 infection was common (*2*). Another explanation for the low level of seroprevalence noted, especially against H5N1, is that some sampling was conducted over summer months, when avian influenza activity is low. Further explanation might include waning antibody titers or lack of seroconversion.

None of the participants had H5N8 antibodies, even though these viruses are not uncommon in poultry in Egypt. In the United States, humans who were exposed to birds infected with clade 2.3.4.4 H5 viruses did not show any acute respiratory symptoms (*22*). Viruses of this clade might not be transmitting efficiently from birds to humans because they have limited capacity for replication and transmission in mammals and mammalian cell lines (*23*).

None of the exposure, health status, or demographic variables we collected were associated with being seropositive. This finding might indicate that being seropositive is related to factors not collected in this study, such as host genetic factors or other behaviors.

Our study had enough power to detect active infection with AIVs. Human infections with H5N1 and H9N2 but not H5N8 were detected. Egypt had previously reported human cases of H5N1 and H9N2 infection; human infection with H5N8 has not been reported. Incidence of infection with any avian influenza virus was 5–18 cases/10,000 poultry-exposed persons, meaning that the number of reported cases is much lower than the number of infections that actually occur. This occurrence can be explained by the fact that none of the case-patients we detected died or required hospitalization, which would make them easily missed by hospital- or clinic-based surveillance. Although the number of reported cases might be underestimated, the reported case-fatality rates are overestimated because of the mild and asymptomatic cases that were missed.

The reported symptoms were similar to symptoms of infection with seasonal influenza viruses. All case-patients had fever, cough, sore throat, and myalgia. Malaise and diarrhea occurred in a few cases. Most case-patients used over-the-counter antipyretic or anti-inflammatory drugs to treat their symptoms. Shedding duration ranged from 1 to 9 days, but no human-to-human transmission was detected, meaning that exposure to viruses that infected the poultry remains the source of human infection. Because of delays in confirming avian influenza in study participants, we were not able to obtain samples from poultry at the time when the participant was ill. Sampling poultry at the same time of sampling humans would have provided better information on how poultry infections correlate with human infections. The exact routes of transmission from poultry to humans, whether direct contact or aerosol, remain to be determined. Observing or documenting behaviors through surveys does not pinpoint transmission routes; hence, future studies should consider measuring individual exposure by using virologic methods, such as determining presence of virus on surfaces or in the air to which humans are exposed, especially in household settings, similar to what has been done in live bird market settings (*24*–*28*).

Serologic and virologic findings were not correlated, especially for H9N2 infections. In follow-up period 1, no cases of H9N2 were detected, yet 11% of the participants had antibodies. In follow-up period 2, cases were detected, but seroprevalence was almost negligible. These findings indicate that relying on serologic tests alone to estimate disease incidence might be misleading, given that many case-patients do not seroconvert, as we found in this study.

In conclusion, backyard poultry growers in Egypt continue to be infected with AIVs that are enzootic in their poultry. To eliminate human cases, poultry infections should be controlled and growers’ awareness increased to decrease the adverse effects of substandard poultry-raising practices.

Appendix Additional information about incidence and seroprevalence of avian influenza in a cohort of backyard poultry growers, Egypt, August 2015–March 2019.
