# Supplementary material for: Incidence and Seroprevalence of Avian Influenza in a Cohort of Backyard Poultry Growers, Egypt, August 2015–March 2019
Source: Emerg Infect Dis. 2020 Sep;26(9):2129–36. doi: 10.3201/eid2609.200266 (PMC7454077; doi:10.3201/eid2609.200266)
Supplement: Appendix — Additional information about incidence and seroprevalence of avian influenza in a cohort of backyard poultry growers, Egypt, August 2015–March 2019. [file 20-0266-Techapp-s1.pdf]

Article DOI: https://doi.org/10.3201/eid2609.200266

# Incidence and Seroprevalence of Avian Influenza in a Cohort of Backyard Poultry Growers, Egypt, August 2015–March 2019

## Appendix

**Appendix Table.** Description of households enrolled in the study

| Governorate | No. households | No. subjects (%) | Median no. subjects per household (range) |
|---|---|---|---|
| Fayyoum | 86 | 480 (20.0) | 5 (2–17) |
| Kafr El Sheikh | 85 | 542 (22.6) | 5 (1–20) |
| Qalyubiya | 81 | 486 (20.2) | 5 (1–20) |
| Gharbiya | 60 | 321 (13.4) | 5 (1–14) |
| Sharkiya | 78 | 573 (23.9) | 5 (1–20) |

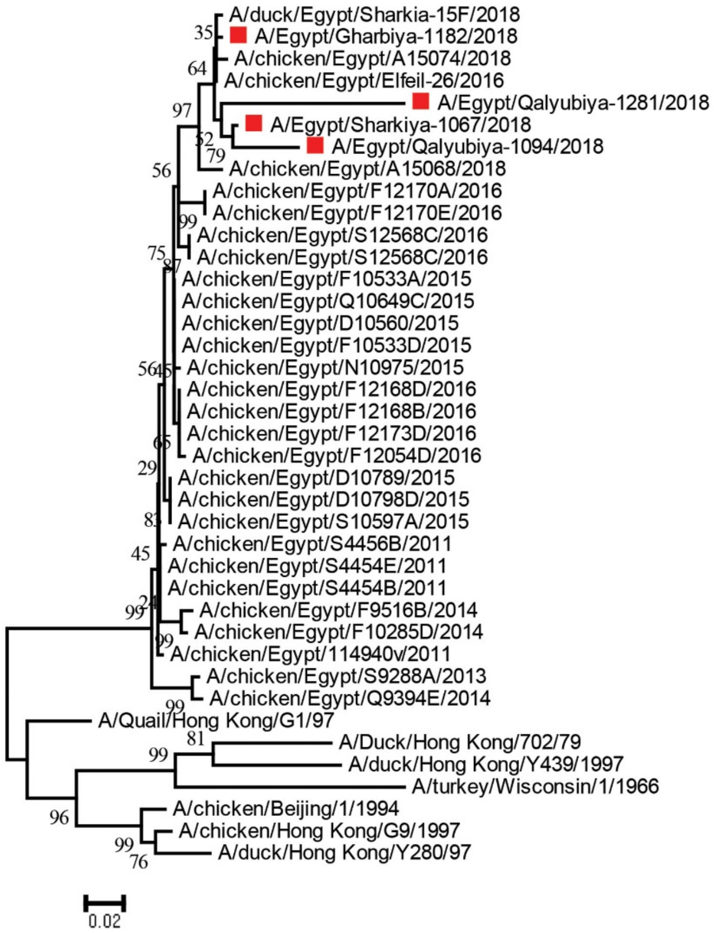


**Appendix Figure 1.** Phylogenetic analysis of the hemagglutinin gene of H9N2 viruses from infected cases (red squares). Those viruses belonged to G1-like viruses endemic in Egypt

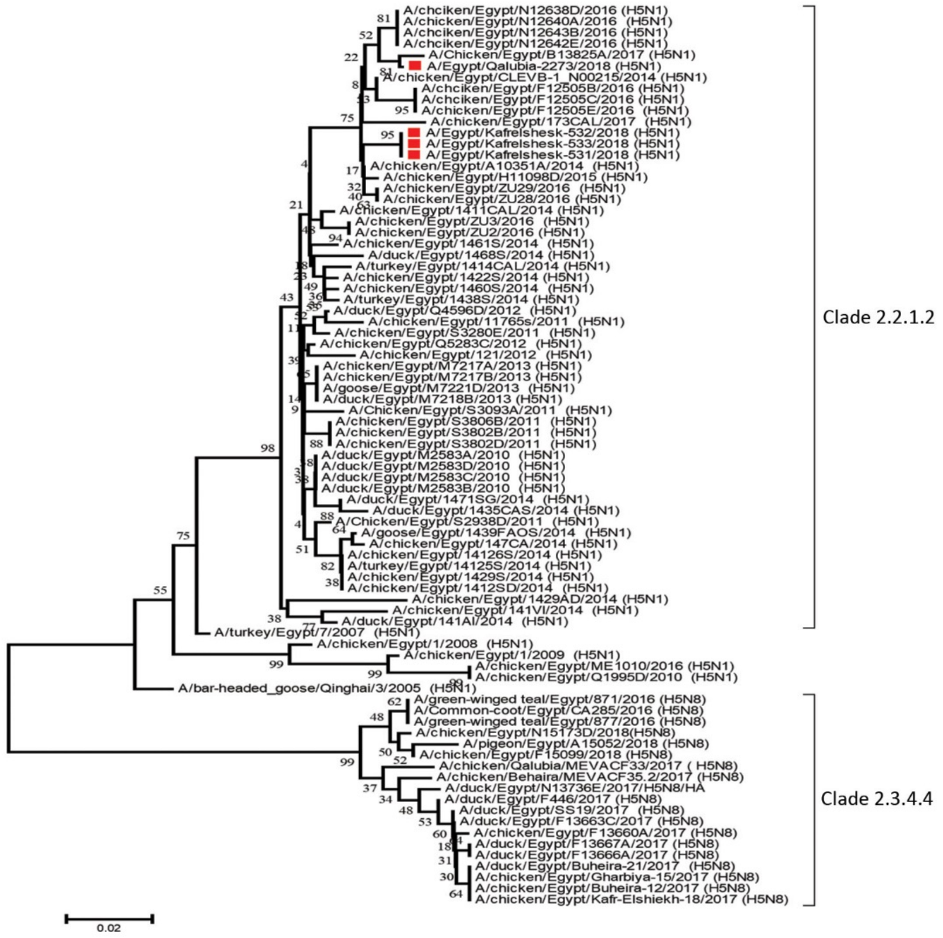


**Appendix Figure 2.** Phylogenetic analysis of the hemagglutinin gene of H5N1 viruses from infected cases (red squares). Those viruses belonged to clade 2.2.1.2 viruses endemic in Egypt
